# Measuring the intensity of mental healthcare: development of the Mental Healthcare Intensity Scale (MHIS)

**DOI:** 10.1186/s12888-023-04752-6

**Published:** 2023-05-30

**Authors:** Thijs Beckers, Bauke Koekkoek, Bea Tiemens, Giel Hutschemaekers

**Affiliations:** 1 Primary Healthcare Department, MET ggz, Minister Beverstraat 3, Roermond, 6042 BL The Netherlands; 2 Research Group in Social Psychiatry and Mental Health Nursing, HAN University of Applied Science, Nijmegen, The Netherlands; 3 Behavioural Science Institute, Radboud University, Nijmegen, The Netherlands; 4 Pro Persona Research, Renkum, The Netherlands; 5 Indigo, Utrecht, The Netherlands

**Keywords:** Mental health services, Intensity of care, Assessment instrument

## Abstract

**Background:**

There are considerable differences among mental healthcare services, and especially in developed countries there are a substantial number of different services available. The intensity of mental healthcare has been an important variable in research studies (e.g. cohort studies or randomized controlled trials), yet it is difficult to measure or quantify, in part due to the fact that the intensity of mental healthcare results from a combination of several factors of a mental health service. In this article we describe the development of an instrument to measure the intensity of mental healthcare that is easy and fast to use in repeated measurements.

**Methods:**

The Mental Healthcare Intensity Scale was developed in four stages. First, categories of care were formulated by using focus group interviews. Second, the fit among the categories was improved, and the results were discussed with a sample of the focus group participants. Third, the categories of care were ranked using the Segmented String Relative Rankings algorithm. Finally, the Mental Healthcare Intensity Scale was validated as a coherent classification instrument.

**Results:**

15 categories of care were formulated and were ranked on each of 12 different intensities of care. The Mental Healthcare Intensity Scale is a versatile questionnaire that takes 2-to-3 min to complete and yields a single variable that can be used in statistical analysis.

**Conclusions:**

The Mental Healthcare Intensity Scale is an instrument that can potentially be used in cohort studies and trials to measure the intensity of mental healthcare as a predictor of outcome. Further study into the psychometric characteristics of the Mental Healthcare Intensity Scale is needed.

**Supplementary Information:**

The online version contains supplementary material available at 10.1186/s12888-023-04752-6.

## Introduction

Services for people with mental health problems are delivered in many ways. They vary considerably among different communities, regions, and nations. In developed countries a substantial number of different services are available, which adds to the difficulty of distinguishing between the content and intensity of the services. The intensity of mental healthcare is an important variable in cohort studies and randomized controlled trials, yet it is difficult to measure and quantify. For example, when evaluating the long-term effects of an intervention, it is essential that the intensity of the care be measured in order to rule out changes in the mental healthcare provision as a confounding variable.

The concept of intensity of mental healthcare is a combination of several factors: (a) the frequency and duration of contact with mental healthcare professionals, (b) the type of mental healthcare (general vs. specialist or nursing vs. psychological interventions), (c) the location of the care that is provided, (d) the educational level of the healthcare professionals, (e) whether there are other patients receiving the same treatment (e.g. group therapy), and (f) whether there is additional mental healthcare (e.g. medication) [1]; [2]; [3]; [4]. There are instruments and questionnaires (a) aiming at concepts related to the intensity of mental healthcare, e.g. the cost of mental health problems [5], (b) aimed at a specific situation or study [6]; [7], or (c) based on the mapping of a complete healthcare system [8]; [9]. None of these, however, provides a metric for the intensity of the mental healthcare that can easily be used in mental healthcare research or clinical practice [10]; [11].

The Care Content Checklist (CCCL) is, to our knowledge, the only instrument that measures the intensity of mental healthcare on an individual level [12]. The CCCL, however, has a serious drawback in that it takes a research assistant approximately 20 min to complete it with each participant. Additionally, there is no method for reducing the 34 variables that the CCCL measures into a useful score, thereby rendering the CCCL as not particularly user-friendly.

In this paper, we describe the development of an instrument for measuring the intensity of mental healthcare that is based on the CCCL. In developing the new instrument, we aimed to ensure that it was user-friendly. Our goals were to develop the instrument as a self-report questionnaire, to reduce the time required to complete the instrument as much as possible, and to develop a method for easily deriving a single outcome scale that can be used in future studies.

## Methods

The MATCH cohort study was a four-year multicentre naturalistic cohort study, which included yearly assessments [12]. Participants in the MATCH cohort study were recruited from different parts of the mental healthcare system in the Netherlands. The study encompassed both cities and rural areas, and included primary, secondary and tertiary provisions in three Dutch mental health services. One of the instruments used in the MATCH cohort study was the CCCL, which was referred to earlier. The CCCL is a 34-item questionnaire, which can be administered by a research assistant. It collects information about the four aspects of mental healthcare described earlier, including the number of sessions attended per year and the approach taken in the treatment or the type of residential mental healthcare. Although the CCCL collects a complete set of information, no method has been developed to condense the information into a single variable that can be used for analysis. This study, therefore, was designed to reduce the number of variables and to develop a method for deriving a single score from the reduced set of variables. It did so by first establishing frequently occurring categories of mental healthcare and then ranking these categories.

The wide range of different types of adult mental healthcare provided by mental health services was a hurdle that had to be surmounted in order to develop an instrument for measuring the intensity of mental healthcare in general. In order to disentangle the nuances among the different interventions, we first of all had to distinguish between the intensity of mental health services based on their location. We did so by identifying four different types of mental health services [13]; [8]. Building on the distinction based on location, we could then identify four types of mental health services. The first and most common type is outpatient mental health services. It is any mental health service for which the patient must travel to the location where the service is being provided in order to have a session with a healthcare professional. The second type of mental healthcare is delivered in the patient’s home. It is similar to outpatient mental healthcare, but the healthcare professional must travel to the patient’s home. The third type of mental healthcare is day treatment, which often takes place separately from other mental health services. It includes group activities, although often not exclusively, and these can be either occupational or have a more therapeutic focus. The fourth and last type of mental health service is residential mental healthcare, which usually takes place in supported housing facilities or in a psychiatric hospital (or psychiatric ward in a general hospital).

Another issue to consider when operationalizing the intensity of mental healthcare is the variety in mental healthcare services across different countries. There are wide variations in the pathways for accessing mental healthcare, the levels of cooperation among healthcare services, and the general availability of psychiatric hospitals, and these differences are only exacerbated when we consider middle- and low-income countries [14]; [15]; [16]; [10]; [17]. As a consequence of these differences, we aimed to develop a questionnaire that was general enough to encompass all types of mental health services, but at the same time precise enough to provide a meaningful classification system, especially when used for research purposes.

**Fig. 1:**
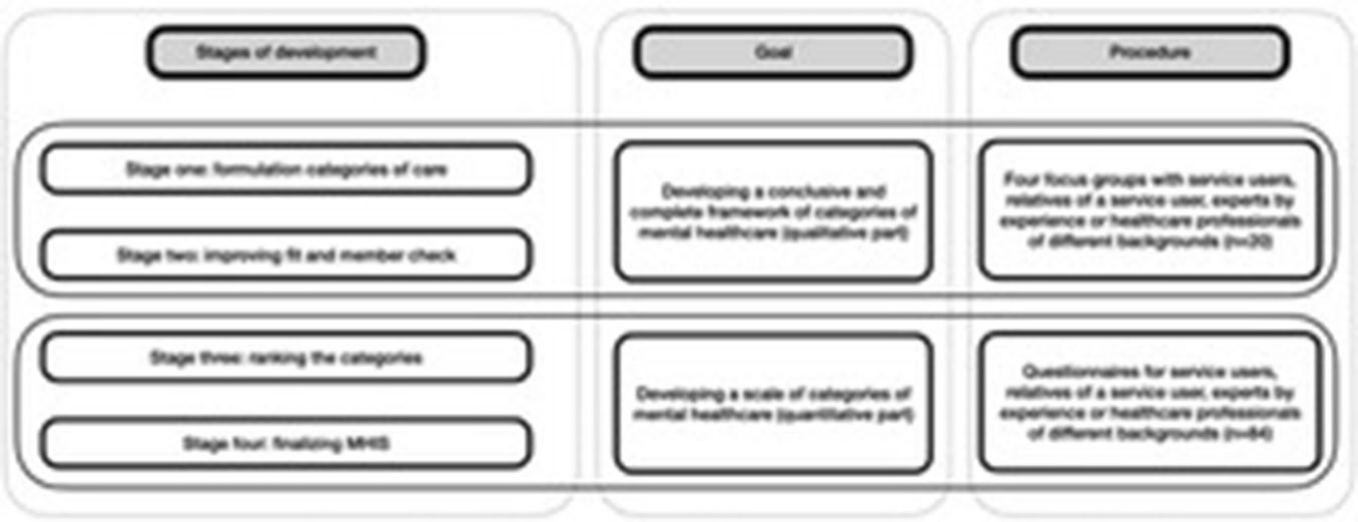
Overview of the developmental process

This instrument for measuring intensity of mental healthcare was developed in four stages: (1) the categories of care were formulated, (2) the fit among the categories of care was improved, (3) the categories of care were rank-ordered, and (4) the Mental Healthcare Intensity Scale (MHIS) was finalized as an instrument that coherently classifies the intensity of mental healthcare. An overview of the developmental process is shown in Fig. 1. Two samples of participants were recruited for the study. For Stage One and Stage Two, people were recruited who were involved in mental healthcare (service users, relatives of service users, experts by experience, and mental health professionals). For Stage Three and Stage Four, we recruited separate service users, relatives of service users, and mental health professionals to complete the questionnaire.

### Stage one: formulation categories of care

The aim of the first stage was to formulate a limited number of categories of care based on participants’ knowledge about the organization of mental healthcare. We considered it important to keep the matrix of the categories understandable and accessible. Thus, after deliberating we decided on a maximum of four categories for each of the four areas of mental healthcare, for a total maximum of 16 categories. Because few people are knowledgeable about the entire mental healthcare system, four different focus group meetings were organized for the four different types of mental healthcare: outpatient mental healthcare, mental healthcare at home, day treatment in mental healthcare, and residential mental healthcare (including treatment in psychiatric hospitals). Five participants were invited for each focus group meeting, with a mixture of the following people with these roles: service user, relative of a service user, expert by experience, and mental healthcare professional. These roles were chosen for their potentially different views about mental healthcare. The participants were recruited from the researchers’ networks. All the participants had to be familiar with the type of the mental healthcare services that was discussed in the particular focus group meeting, and at least 40% of the participants had to be mental healthcare professionals. Because of COVID-19 restrictions during this stage of the study, the focus group interviews were conducted using video conferencing. Each focus group meeting was led by two researchers. One researcher guided the group process, whereas the other one oversaw the subject that was being discussed. To facilitate analysis of the results, all of the focus group meetings were video- and audio-recorded.

Nominal Group Technic [18] was used to explicate the participants’ knowledge of mental healthcare and to achieve a consensus on its categories. Each focus group meeting included five steps. In the first step, the characteristics of the different categories of mental healthcare were discussed, with the items on the Care Content Checklist (CCCL) being used as in the MATCH cohort study [12] as input for starting the discussion. In the second step, the participants had five minutes to determine for themselves which three characteristics of mental healthcare they considered the most important regarding the intensity of care. In Step Three they were asked to assign one point to each of the three most important characteristics. In Step Four the characteristics of mental healthcare that had received the most points from the participants were discussed (along with the number of points each characteristic had received). In the fifth and final step decisions were made about the most important characteristics of mental healthcare, thereby reaching consensus on 2-to-4 different categories of mental healthcare within the scope of the type of mental healthcare discussed at that specific focus group meeting.

### Stage two: improving the categories and confirming members’ agreement

In the second stage the categories were combined that had been agreed during the four focus group interviews (Stage One) and the coherence in language use was improved and criteria were set. In this stage the researchers formulated the categories based on the consensus that had been reached during the four focus group meetings, and an easy-to view table with all of the categories was constructed. Special attention was paid to creating a comprehensive table of the categories, so that each form of mental healthcare could be placed into a specific category. The criteria for each category prevented assignment to a second category in order to avoid overlap. The table was then shown individually to five participants in the focus group meetings for each member’s confirmation of its accuracy [19]. Participants in each of the focus groups with all roles from each area of mental healthcare were included in the members’ check.

### Stage three: ranking the categories

In the third stage, the categories of mental healthcare that were formulated in Stage One and Stage Two were rank-ordered according to the intensity of care that each category provided. For this exercise, service users, relatives of service users, and mental healthcare professionals were recruited from the researchers’ networks. The authors asked acquaintances who matched the criteria to participate. Additionally, they enlisted the help of colleagues in order to include enough participants who met the criteria. To ensure that a representative sample was achieved, attention was paid to the role of the participants in mental healthcare services (e.g. service user, healthcare professional) and to the relationship of the participants to the different areas of mental healthcare (outpatient mental healthcare, home mental healthcare, day treatment, and residential mental healthcare). Participants in all roles and areas of mental healthcare were included in the final sample. Because a larger sample than in Stage One and Stage Two resulted, we differentiated less among the different roles than in Stage One and Stage Two. None of the participants in Stage One or Stage Two were included in Stage Three and Stage Four.

These participants were asked to rank the categories of mental healthcare according to the intensity of care that each provided. To ease the work of the participants and to ensure their accuracy in ranking the 15 categories (while providing an explanation for each category), were randomly divided into three groups of five each for each of the participants [20].

### Data analysis

An automated algorithm for processing the input data for Segmented String Relative Rankings (SSRRs) was used to rank order each of the categories according to each participant’s input [21]. The SSRR methodology and algorithm were developed for ranking units based on surveys of expert opinions. It is based on link analysis, which is a common form of analysis in network theory that is used to evaluate relationships among units. The algorithm treats data as part of a linear, hierarchical network, and each unit receives a score according to how many units are positioned below it in the network. Therefore, each unit has an individual score that indicates its rank relative to the other units. Units with higher scores (indicating less intensive care) are positioned higher in the network than units with lower scores. The approach of ranking that is based on the accumulated scores of each individual unit makes it efficient for resolving contradictions among experts and by providing a clear ranking as long as there is sufficient input of data. The algorithm also indicates when there is too little data to reliably distinguish specific categories from one another. The code used for the present analysis is included in the supplements.

In addition to using the standard SSRR methodology and algorithm, differences among the individual categories were also evaluated. With sufficient data, the SSRR algorithm can reliably detect the smallest differences among items and rank them accordingly. However, the smallest differences may not be meaningful, so that it is necessary to interpret such automatically distinct differences using authentic and meaningful explanations [22]. Unfortunately, to our knowledge, there is no established method for assessing a minimal clinically relevant difference among different forms of healthcare. Therefore, it was necessary to explore other ways to determine the minimal clinically relevant difference. When the difference between two categories of mental healthcare was less than 2% of the total difference between the least and most intensive categories of mental healthcare, the categories were treated as equally intensive. Thus, although categories might differ statistically, they were not considered sufficiently different in clinical practice and thus were not assigned different levels in the MHIS.

Finally, a subgroup analysis was performed for (a) service users and their relatives and (b) healthcare professionals. The descriptive statistics were analysed using JASP open-source software for statistical analyses.

### Stage four: finalizing the MHIS

In the fourth and last stage of formulating the categories, the rankings together with the criteria for assigning them were combined into the final version of MHIS. A schematic decision tree for use in research was also included.

### Ethical considerations

Because this study only asked participants about their views on a specific topic and did not include any data from patients, a formal review by an ethics committee was not needed according to either professional organizations or Dutch law. Nevertheless, the study was reviewed and approved by the scientific committee of the main participating mental health service. During all stages of the study, all participants gave informed consent prior to their participation. Additionally, all procedures used in the study complied with the ethical standards of the Helsinki declaration of 1975, as revised in 2013.

## Results

The steps designed to develop the CCCL into a new instrument for measuring the intensity of mental healthcare were executed in line with the procedure outlined earlier.

### Stage one: formulation of categories of care

In Stage One, the participants in the four focus group meetings met the criteria that had been established beforehand (see Table 1).

**Table 1:** Participants in Stage One and Stage Two (n = 20)

Characteristic	Data	
Gender	Female	13 (65%)
	Male	7 (35%)
Age		Mean = 39.0 (SD = 11.5)
Role (more than one option possible per participant)	Service user	7
	Relative of a service user	2
	Expert by experience	4
	Mental healthcare professional	13
Speciality of the healthcare professionals	Psychiatrist	2
	Nurse	4
	Advanced practice nurse	1
	Psychologist	3
	Social worker	3
Years involved in mental healthcare		Mean = 15.2 (SD = 12.8)

In the first focus group meeting, which lasted 40 min, outpatient mental healthcare was discussed. The participants quickly identified two distinguishing features of outpatient mental healthcare: the frequency of sessions and the amount of additional care (e.g. medication, group sessions, art therapy, physical therapy, sheltered work). There was then a discussion of how to limit the number of categories of outpatient mental healthcare to four (the maximum number of categories of each type of mental healthcare that had already been established) because of the large number of different treatments that could be named, even after they had been limited to the two distinguishing characteristics identified earlier. After ample deliberation, outpatient mental healthcare was divided into three categories of frequency: fewer than 15 sessions per year, 15-to-51 sessions per year, and 52 or more sessions per year. Additionally, a fourth category was created for service users with 15-to-51 sessions per year but who received additional care (such as medication, group therapy, art therapy, physical therapy, or sheltered work). In the other two categories, no differences were made on the basis of additional care.

The topic of the second focus group meeting (duration of 45 min) was mental healthcare at home. The participants first discussed (a) the least intensive category of care (consisting at most of one home visit per week by a social worker, which was usually aimed at retention of goals already achieved), and (b) the most intensive category of care (daily or near daily home visits by social workers, nurses, and sometimes a psychiatrist, aimed at crisis resolution). Then, the categories of care between these two extremes were determined. The intermediate categories largely resembled the least intensive category of home mental healthcare, but it was aimed more at achieving new goals. The boundaries for the intermediate categories of home mental healthcare were one or more home visits per week and five or more home visits per week.

The topic for the third focus group meeting (duration of 50 min) was day treatment in mental healthcare. The participants discussed how day treatment in mental healthcare differs in both frequency (number of sessions per week) and modality (e.g. psychotherapy versus daytime activities) from other types of mental healthcare. On the basis of these characteristics, they decided to outline three categories of day care: (a) day treatment aimed at daytime activities, (b) psychotherapy at a maximum of three days a week, and (c) psychotherapy more than three days a week. Additional kinds of day care were also discussed but was deemed uncommon in day mental healthcare.

In the fourth and last focus group meeting (duration of 45 min), residential mental healthcare was discussed. The participants quickly agreed that supported housing should be one of the categories of residential mental healthcare. Thereafter, the different kinds of admissions to a psychiatric hospital were discussed. One key type of residential care was treatment on a crisis ward aimed at stabilizing the service user or on a ward aimed at long-term treatment, and the second most important feature was whether the admission was to an open or closed ward. This discussion resulted in a consensus being reached on three other categories of residential mental healthcare: (a) long-term inpatient treatment, (b) admission to an open crisis ward, and (c) admission to a closed crisis ward. The participants agreed that additional care was non-existent for patients who had already been admitted, but it did occur occasionally for patients in supported housing. This, however, was not common enough to warrant its being included as a separate category. Supported housing, moreover, would in itself almost always be the most intensive form of mental healthcare.

### Stage two: improving the categories and confirming members’ agreement

Five participants in the focus group meetings completed a member’s check: (a) the psychiatrist who participated in the focus group meeting on residential mental healthcare, (b) the expert by experience who participated in the focus group meeting on day mental healthcare, (c) the social worker who participated in the focus group meeting on mental healthcare at home, (d) the psychologist who participated in the focus group meeting on outpatient mental healthcare, and (e) the service user who participated in the focus group meeting on mental healthcare at home. Following these members’ checks, the language used in the 3 of the 15 categories of mental healthcare was altered. Additionally, in the category of day mental healthcare at home, it was specified more explicitly that the treatment should be multidisciplinary. No further remarks or changes were made in these categories. After the members’ check, the categories of care were considered to be final (see Table 2).

**Table 2:** Categories of mental healthcare

Outpatient mental healthcare	Mental healthcare at home	Day treatment in mental healthcare	Residential mental healthcare
Short-term or low intensity outpatient mental healthcare: either short or low intensity mental healthcare aimed at improving mental health. Criteria: Outpatient mental healthcare is the most important form of mental healthcare. It includes14 or fewer sessions per year.	Low intensity mental healthcare at home: sessions aimed at retention of already achieved goals. Criteria: It is the most important form of mental healthcare. 1 session or less per week by a social worker.	Daytime activities: day treatment aimed at providing activities, usually 1-to-4 days a week, sometimes complimented by group training. Criteria: Day treatment in mental healthcare is the most important form of mental healthcare. Aims to provide daytime activities.	Supported housing: long-term living, usually located in a residential neighbourhood. Criteria: Residential mental healthcare is the most important form of mental healthcare. The unit/ward aims to keep patients > 2 years.
Outpatient mental healthcare: sessions aimed at improving mental health. It is the most important form of mental healthcare and includes 15 or more, but fewer than 52 sessions per year. No more than 1 type of additional mental healthcare (e.g. medication, group sessions, art therapy, physical therapy. or sheltered work)	Average intensity mental healthcare at home; sessions aimed at improving mental health. Criteria: Mental healthcare at home is the most important form of mental healthcare. More than 1 but less than 4 sessions per week by a social worker.	Low intensity day treatment. Psychotherapy aimed at improving mental health during a maximum of three days per week. Criteria: Day treatment in mental healthcare is the most important form of mental healthcare. Provides psychotherapy three days per week or less.	Long-term admission (6–24 months) aimed at improving mental health. Criteria: Residential mental healthcare is the most important form of mental healthcare. The unit/ward aims to retain patients in treatment 6-to-24 months.
Outpatient mental healthcare with additional care: sessions aimed at improving mental health. It is the most important form of mental healthcare and includes 15 or more, but fewer than 52 sessions per year and 2 or more types of additional mental healthcare (e.g. medication, group sessions, art therapy, physical therapy. or sheltered work)	High intensity mental healthcare at home: sessions aimed at improving mental health. Criteria: Mental healthcare at home is the most important form of mental healthcare. 4 or more sessions per week by a social worker.	High intensity day treatment: psychotherapy aimed at improving mental health during four or more days per week. Criteria: Day treatment in mental healthcare is the most important form of mental healthcare. Provides psychotherapy four or more days per week	Admission on an open crisis ward of a psychiatric hospital aimed at stabilizing acute mental illness. Criteria: It is the most important form of mental healthcare. The unit/ward aims to retain patients less than 6 months in treatment. The ward is open
High intensity outpatient mental healthcare: multiple sessions per week aimed at improving mental health. Outpatient mental healthcare is the most important form of mental healthcare. It includes 52 sessions or more per year.	Acute mental healthcare at home: sessions aimed at treating acute mental illness. Criteria: It is the most important form of mental healthcare. Sessions by a multidisciplinary team (e.g. nurses, social workers, and a psychiatrist).		Admission on a closed crisis ward aimed at stabilizing mental illness Admission on an open ward in a psychiatric hospital to treat acute mental illness. Criteria: Residential mental healthcare is the most important form of mental healthcare. The unit/ward aims to retain patients less than 6 months in treatment. The ward is closed

### Stage three: ranking the categories

All of the participants in Stage Three were confirmed to be either service users, a relative of a service user, or a mental healthcare professional (see Table 3).

**Table 3:** Participants in Stage Three (n = 84)

Characteristic	Data	
Gender	Female	55 (65%)
	Male	28 (33%)
	Other	1 (1%)
Age		Mean = 41.3 (SD = 12.4)
Involvement in mental healthcare (in years)		Mean = 16.0 (SD = 10.7)
Role (more than one option possible per participant)	Service user	16 (19%)
	Relative of a service user	15 (18%)
	Mental healthcare professional	53 (63%)
Specialty of the healthcare professionals	Psychiatrist	7 (13%)
	Nurse	17 (31%)
	Social worker	12 (22%)
	Psychologist	15 (28%)
	Expert by experience	3 (6%)
	Other	1 (2%)
Educational level of the healthcare professionals	Bachelor’s degree or less	20 (37%)
	Master’s degree or higher	34 (63%)

The SSRR algorithm provided a clear ranking of the categories of mental healthcare that could statistically and reliably be differentiated (see Table 4). The difference between the lowest (most intensive) and the highest (least intensive) categories was 494 points, and the difference between adjacent categories ranged from 17 to 62 points. The scores that the SSRR algorithm produces are not comparable across different applications of the algorithm because they are a product of the number of items and the spread between the items.

**Table 4:** Ranking of the categories of mental healthcare according to the SSRR algorithm

Ranking according to SSRR	Item	Value	Delta
1	Low intensity mental healthcare at home	1856	-
2	Short or low intensity outpatient mental healthcare	1839	17
3	Outpatient mental healthcare	1816	23
4	Average intensity mental healthcare at home	1813	3
5	Daytime activities	1807	6
6	Low intensity day treatment	1804	3
7	Supported housing	1748	56
8	High intensity day treatment	1696	52
9	High intensity mental healthcare at home	1675	21
10	High intensity outpatient mental healthcare	1649	26
11	Acute mental healthcare at home	1621	28
12	Outpatient mental healthcare with additional care	1612	9
13	Long-term admission	1446	166
14	Admission to an open crisis ward	1424	22
15	Admission to a closed crisis ward	1362	62

There were, however, two exceptions. The first was the difference between the three inpatient treatment categories and the other categories, which was 166 (34% of the total difference). Secondly, there were five categories that differed by a small amount (< 2% of the total difference) from another category. As detailed in the Methods section, these categories were given equal rankings. The final ranking of the categories of mental healthcare used in the MHIS is displayed in Table 5.

**Table 5:** Final ranking of categories of care in the MHIS

Category	Final ranking
No mental healthcare (except for care provided by a general practitioner)	0
Low intensity mental healthcare at home	1
Short or low intensity outpatient mental healthcare	2
Outpatient mental healthcare	3
Average intensity mental healthcare at home	3
Daytime activities	3
Low intensity day treatment	3
Supported housing	4
High intensity day treatment	5
High intensity mental healthcare at home	6
High intensity outpatient mental healthcare	7
Acute mental healthcare at home	8
Outpatient mental healthcare with additional care	8
Long-term admission	9
Admission to an open crisis ward	10
Admission to a closed crisis ward	11

In the subgroup analysis, both the group called *service users and their relatives* and the group called *mental healthcare professionals* deviated somewhat as shown in Table 5. However, these deviations were for categories of care that were merged because of the small differences between them and other categories, which justified the need to merge them with other categories of care.

### Stage four: finalizing the MHIS

**Fig. 2:**
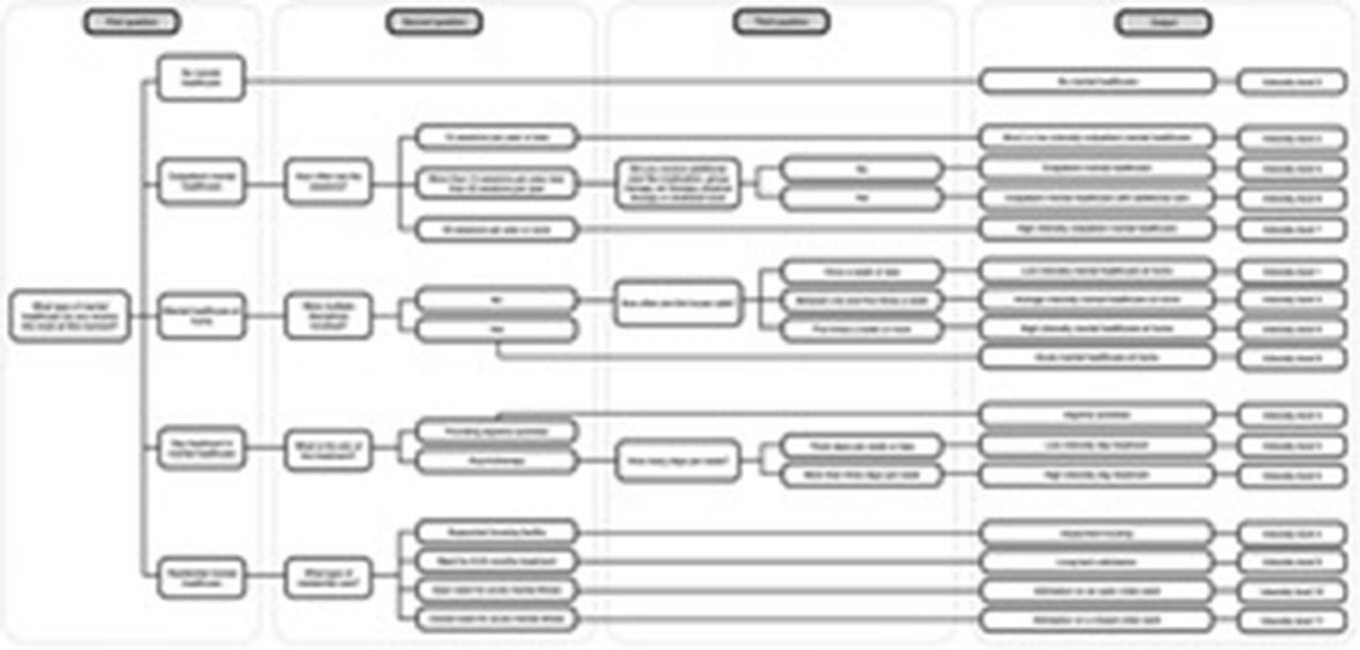
Scheme of adaptive questions in the MHIS

When the categories and ranking were combined, the version of the MHIS that resulted included 2-to-3 questions in an adaptive scheme (see Fig. 2) that can be used to allocate a participant to a specific intensity of mental healthcare. The MHIS can be completed either as a self-report or during an interview, which requires only 1-to-2 min as there are only 2-to-3 questions to ask the respondent.

## Discussion

We developed an instrument for assessing the intensity of mental health services. The aims were (a) for people either receiving or giving professional help to be able to easily complete it, and (b) for the results to be readily usable in statistical analysis. The four-stage developmental process resulted in the Mental Healthcare Intensity Scale (MHIS). It yields both a category of care and an intensity of care that can range from *0* (*no mental healthcare*) to *11* (admission to a closed ward for crisis resolution). The MHIS can be administered either online or in a paper format, and it takes a participant only 1-to-2 min to complete without the help of a research assistant. Alternatively, the MHIS can be administered as a short interview. Because of the short completion time, the MHIS is especially suitable for use in repeated administrations, for example in cohort studies or randomized trials.

As in all questionnaires and other measuring instruments, it is important to define the target group [23] for whom the MHIS is intended. As indicated, the MHIS is intended for use by all people who either receive or provide mental healthcare. The MHIS is based on the Care Content Checklist (CCCL), which asks patients about all mental healthcare they receive. Accordingly, the CCCL was used in Project MATCH to collect information about the frequency and type of mental healthcare from participants with diverse needs for mental health services [12]. Similarly, the MHIS is suitable for use with all people who receive mental healthcare.

The MHIS asks participants about the mental healthcare they received during the past year. A possible concern, therefore, is that respondents’ memory might be fallible across this relatively long period of time. Recall biases might occur, especially when the MHIS is used in studies aimed at identifying the causes of a diseases. For instance, affected participants might have an incentive to spend more time and effort searching their memory than control participants [24]. Having a recall bias is, however, different than simply not being able to recall the requested information. Such a memory failure is likely to affect all participants equally [25]; [26]. It is important, therefore, to distinguish between inaccurate recall and biased recall [26]. There are, however, ways to limit recall bias when the MHIS is being used, e.g. by not informing the participants of the exact hypotheses being tested [27]; [28] and not to extend the time space more than is necessary that the MHIS is intended to measure [29]. Recall bias, therefore, does not have to be a problem when the MHIS is being used; nevertheless, it should always be taken into consideration, especially when diverse groups of participants are being compared.

The MHIS assesses type and intensity of mental healthcare as provided in most healthcare systems in western countries. This independence from specific healthcare systems was achieved by basing the MHIS on existing information about healthcare systems around the world [8]; [9] and by including knowledgeable participants in all stages of the developmental process. Nevertheless, the MHIS does not adequately represent all possible mental healthcare options and systems around the world. The lack of representativeness applies especially to middle- and lower-income countries and to new and innovative interventions, such mental healthcare that is being offered through virtual reality. We suggest, therefore, that the initial version of the MHIS be viewed as a dynamic model for measuring the intensity of care. It is intended for use in most western healthcare systems, and it can be adapted for use in other healthcare systems or updated to include innovative interventions in mental healthcare.

## Strengths and weaknesses

The thorough developmental process which uses the insights of experts that are ranked using sophisticated analysis is an apparent strength of the MHIS. It has contributed to the validity of the MHIS as a measuring instrument. On the other hand, there is limited knowledge on the psychometric characteristics of the MHIS as a newly developed instrument. Test-retest reliability or inter-rater reliability, for example, need to be studied in the future in order to fully evaluate the MHIS as an instrument. Additionally, the risk of recall bias is a possible weakness, especially when insufficient care is given to considering the influence of recall bias.

## Conclusions

The MHIS is an instrument that can potentially be used to measure the intensity of mental healthcare in scientific studies. The MHIS takes only a few minutes to complete and produces a single outcome score. However, additional research, especially on the psychometric properties of the MHIS, is needed.

## Electronic supplementary material

Below is the link to the electronic supplementary material.

Supplementary Material 1 Segmented String Relative Rankings algorithm

## Data Availability

The datasets generated and analysed during Stage One of the current study are not publicly available because it was not possible to conceal the identity of the participants in the focus group interviews. The data are, however, available from the corresponding author on reasonable request. The datasets that were generated and analysed during Stage Three of the current study are available in the Figshare repository, https://doi.org/10.6084/m9.figshare.21564435.v1.

## Abbreviations

MHIS: Mental Healthcare Intensity Scale
SSRRs: Segmented String Relative Rankings
CCCL: Care Content Checklist
