# Supplementary material for: Measuring the intensity of mental healthcare: development of the Mental Healthcare Intensity Scale (MHIS)
Source: BMC Psychiatry. 2023 May 30;23:377. doi: 10.1186/s12888-023-04752-6 (PMC10227812; doi:10.1186/s12888-023-04752-6)
Supplement: Supplementary file 1 — Supplementary Material 1 Segmented String Relative Rankings algorithm [file 12888_2023_4752_MOESM1_ESM.docx]

<?php

header('Content-Type:text/html; charset=utf-8');

$damping = 0.85;

$units = array();

$experts = array();

$ranking = array();

$segments = array();

**if**(($handle = fopen('/Users/thijsbeckers/Desktop/Data MHIS stap 4/naas.csv', 'r')) !== **false**) {

**while**(($data = fgetcsv($handle, 0, ",")) !== **false**)

{

$units[] = trim($data[0]);

$experts[] = trim($data[1]);

$ranking[] = trim($data[2]);

unset($data);

}

fclose($handle);

}

$numberOfOservation = sizeof($units);

**foreach** (array_unique($experts) as $e) {

$segments[$e] = array();

}

**foreach** ($units as $k => $v) {

$segments[$experts[$k]][$v] = $ranking[$k];

}

**foreach** ($segments as $k => $v) {

asort($v);

$segments[$k] = $v;

}

$units = array_values(array_unique($units));

function findChildren($parent, $unit, $children, $level) {

global $segments;

global $damping;

$generation = array();

**foreach**($segments as $segment) {

**if** (isset($segment[$unit])) {

$unitRank = $segment[$unit];

**foreach**($segment as $pk => $pv) {

**if** ($pk != $parent && $pv > $unitRank) {

**if** (isset($children[$pk])) {

$children[$pk] = $children[$pk] + 1 * pow($damping, $level);

} **else** {

$children[$pk] = 1 * pow($damping, $level);

$generation[] = $pk;

}

}

}

}

}

**if** (sizeof($generation) > 0) {

$level++;

**foreach**($generation as $child) {

$children = findChildren($parent, $child, $children, $level);

}

}

**return** $children;

}

$rank = array();

**foreach**($units as $unit) {

$children = array();

$rank[$unit] = array_sum(findChildren($unit, $unit, $children, 0));

}

arsort($rank);

$result = array();

**foreach** ($rank as $k => $v) {

$result[$v][] = $k;

}

$numberOfSegments = sizeof($segments);

$numberOfUnits = sizeof($units);

$notice = **false**;

$i = 1;

$delta = 0;

echo "<style>table, th, td { border: 1px solid black; border-collapse: collapse; } th, td { padding: 5px; }</style>";

echo "<h4>Ranking for ". $numberOfOservation ." observations of " . $numberOfUnits . " units in " . $numberOfSegments . " segments (damping factor = " . $damping . "):</h4>";

echo "<table border=1>";

echo "<tr>";

echo "<th>Ranking</th><th>Item</th><th>Value</th><th>Delta</th>";

echo "</tr>";

echo "<tr>";

**foreach** ($result as $k => $v) {

**if** ($delta != 0) {

$delta = $delta - $k;

}

**if** ( sizeof($v) > 1) {

$notice = **true**;

echo "<tr>";

echo "<td>".$i."</td>";

echo "<td style='color: red;'>";

**foreach**($v as $vk => $vv){

echo (0 == $vk) ? $vv : " | " . $vv;

}

echo "</td>";

echo "<td>".$k."</td>";

echo (0 == $delta) ? "<td>-</td>" : "<td>" . $delta . "</td>";

echo "</tr>";

} **else** {

echo "<tr>";

echo "<td>".$i."</td>";

echo "<td>" . $v[0] . "</td>";

echo "<td>".$k."</td>";

echo (0 == $delta) ? "<td>-</td>" : "<td>" . $delta . "</td>";

echo "</tr>";

}

$delta = $k;

$i++;

}

echo "</tr>";

echo "</table>";

**if** ($notice) {

echo "<h2>Notice:</h2><hr><p>The above list conatins units with equal ranking (colored red). These units have the same position in the final list. Please, consider providing extra data with ranking of these units relative to each other.</p><hr>";

}

?>
